# The Role of the Two-Component QseBC Signaling System in Biofilm Formation and Virulence of Hypervirulent *Klebsiella pneumoniae* ATCC43816

**DOI:** 10.3389/fmicb.2022.817494

**Published:** 2022-04-06

**Authors:** Jingnan Lv, Jie Zhu, Ting Wang, Xiaofang Xie, Tao Wang, Zhichen Zhu, Liang Chen, Fengyun Zhong, Hong Du

**Affiliations:** ^1^Department of Clinical Laboratory, The Second Affiliated Hospital of Soochow University, Suzhou, China; ^2^Department of Clinical Laboratory, Suzhou Science and Technology Town Hospital, Suzhou, China; ^3^Hackensack Meridian Health Center for Discovery and Innovation, Nutley, NJ, United States; ^4^Department of Medical Sciences, Hackensack Meridian School of Medicine, Nutley, NJ, United States; ^5^Department of General Surgery, The Second Affiliated Hospital of Soochow University, Suzhou, China

**Keywords:** hypervirulent, *Klebsiella pneumoniae*, QseBC, two-component system, biofilm formation

## Abstract

Hypervirulent *Klebsiella pneumoniae* (hvKP) is an evolving infectious pathogen associated with high mortality. The convergence of hypervirulence and multidrug resistance further challenges the clinical treatment options for *K. pneumoniae* infections. The QseBC two-component system (TCS) is a component of quorum-sensing regulatory cascade and functions as a global regulator of biofilm growth, bacterial motility, and virulence in *Escherichia coli*. However, the functional mechanisms of QseBC in hvKP have not been reported, and we aim to examine the role of QseBC in regulating virulence in hvKP strain ATCC43816. The CRISPR-Cas9 system was used to construct *qseB*, *qseC*, and *qseBC* knockout in ATCC43816. No significant alterations in the growth and antibiotic susceptibility were detected between wild-type and mutants. The deletion of *qseC* led to an increase of biofilm formation, resistance to serum killing, and high mortality in the *G. mellonella* model. RNAseq differential gene expression analysis exhibited that gene-associated biofilm formation (*glgC, glgP, glgA, gcvA, bcsA, ydaM, paaF, ptsG*), bacterial type VI secretion system (*virB4, virB6, virB10, vgrG, hcp*), and biosynthesis of siderophore (*entC, entD, entE*) were significantly upregulated in comparison with the wild-type control. In addition, *qseB*, *ygiW* (encode OB-family protein), and AraC family transcriptional regulator IT767_23090 genes showed highest expressions in the absence of QseC, which might be related to increased virulence. The study provided new insights into the functional importance of QseBC in regulating the virulence of hvKP.

## Introduction

Hypervirulent *Klebsiella pneumoniae* (hvKP) is a gram-negative opportunistic pathogen that has become a worldwide concern due to increasing cases of life-threatening infections in healthy individuals (Harada et al., 2019; Russo and Marr, 2019; Zafar et al., 2019). HvKP is such an invasive strain in part because of its virulence factors that protect it from immunological responses to survive. These virulence factors include capsule, siderophore, fimbriae, biofilm, but the mechanisms of hypervirulence are not fully defined (Choby et al., 2020; Zhu et al., 2021).

Two-component systems (TCS) are dominant bacterial signal-transduction systems (Groisman, 2016). Quorum sensing is a ubiquitous chemical communication process that bacteria use to response to environmental cues (Abisado et al., 2018). The QseBC two-component system is associated with quorum sensing and functions as a regulator of virulence in a wide range of species (Weigel and Demuth, 2016). QseC, a membrane-bound sensor kinase, senses signals (epinephrine/norepinephrine/autoinducer-3) and phosphorylates the response regulator QseB to control the virulence of enterohemorrhagic *Escherichia coli* (EHEC) (Sperandio et al., 2002; Clarke et al., 2006). Activation of QseBC results in enhanced motility and upregulation of flagellar genes in *Salmonella Typhimurium* (Bearson and Bearson, 2008). The *qseC* mutant is attenuated for virulence in rabbits (EHEC) and piglets (*S. Typhimurium*) (Merighi et al., 2009). Likewise, this function of QseBC had been reported in rare species, such as *Edwardsiella tarda*, *Aggregatibacter actinomycetemcomitans*, *Aeromonas hydrophila*. Disruption of *qseB* and *qseC* exhibited impaired flagellar motilities as well as *in vivo* competitive abilities of *Edwardsiella tarda* (Wang et al., 2011). In the oral pathogen *Aggregatibacter actinomycetemcomitans*, activation of QseBC by autoinducer-2 (AI-2) resulted in increased biofilm growth (Novak et al., 2010). Moreover, Khajanchi et al. (2012) found that the decrease in virulence of *Aeromonas hydrophila*Δ*qseB* were associated with reduced production of protease and cytotoxic enterotoxin. However, the regulon and function of QseBC in hvKP is currently unknown. In order to explore the role of QseBC in, *qseB* and *qseC* single and double knock-out mutants of the hvKP strain ATCC43816 were constructed and studied.

## Materials and Methods

### Bacterial Strains, Plasmids, Primers and Growth Conditions

Strains, plasmids and primers used in this study are listed in Supplementary Table 1. The ST439 K2 hypervirulent *K. pneumoniae* ATCC43816 was obtained from the American Type Culture Collection (ATCC). *E. coli* DH5α and *K. pneumoniae* strains were grown aerobically in lysogeny broth (LB) medium (1% tryptone, 0.5% yeast, 1%NaCl). Strains with temperature-sensitive plasmid pCasKP and L-arabinose-inducible expression plasmid pBAD24 were grown at 30°C and in the presence of 0.2% arabinose, respectively. Selective antibiotics were added at the following concentrations: rifampicin 100 μg/ml, apramycin 30 μg/ml, and ampicillin 100 μg/ml.

### Construction of the *qseB, qseC, qseBC* Mutants

The hvKP ATCC43816 *qseB*, *qseC*, and *qseBC* mutants were constructed using the CRISPR/Cas9 mediated genome-editing system (Wang et al., 2018). The plasmid pCasKP was electroporated into ATCC43816, followed by selection on agar containing 30 μg/ml apramycin and PCR confirmation. The 20-nt base-pairing region (N20) of an sgRNA was designed through the online web server.^1^ Primers HindIII-QseB/QseC-N20-For and XbaI-gRNA-Rev were used to amplify the sgRNA fragment from plasmid pSGKP-Rif. The PCR product was subsequently ligated into HindIII and XbaI digested pSGKP-Rif to construct the final pSGKP-Rif with targeted sgRNA (pSGKP-QseB/QseC-N20). We utilized the linear homologous DNA fragment as the repair templates. The linear double-stranded DNA (dsDNA) and the pSGKP-QseB/QseC-N20 plasmid were co-transformed into the L-arabinose-induced pCasKP-positive ATCC43816 to generate the *qseB*, *qseC*, and *qseBC* deletions. The cultures were plated on LB agar plates containing 30 μg/ml apramycin and 100 μg/ml rifampicin, and the mutants were confirmed in selected colonies by both PCR and sequencing.

### Complementation of the *qseC* Mutant

The *qseC* gene was amplified from the chromosomal DNA of ATCC43816 using primers QseC-C-For and QseC-C-Rev. The PCR products were then assembled into the multiple cloning site (MCS) of vector pBAD24 using the ClonExpress ^®^ Ultra One Step Cloning Kit (Vazyme Biotech Co., Ltd., China). The recombinant plasmid pBAD24-*qseC* was transformed into *E. coli* DH5α, and then primers pBAD24-For and pBAD24-Rev were used to confirm positive colonies on LB agar with 100 μg/ml ampicillin. The *qseC* mutant was transformed with the pBAD24-*qseC* plasmid and named as Δ*qseC-*pC*qseC*. ATCC43816 and Δ*qseC* carrying the empty vector pBAD24 were used as control.

### Bacterial Growth Curves

Growth curves of ATCC43816, Δ*qseB*, Δ*qseC*, Δ*qseBC*, ATCC43816-pBAD24, Δ*qseC*-pBAD24, and Δ*qseC*-pC*qseC* were determined by subculturing in LB medium and growth. Briefly, overnight cultures of ATCC43816 and mutants were diluted 1:100 in 20 ml of fresh LB broth and grown at 37°C with shaking at 250 rpm. The cell density was detected per hour by OD_600_ measurements. The experiment was conducted with three independent cultures.

### Antimicrobial Susceptibility Testing

Antimicrobial susceptibility testing was performed using a BD Phoenix™ 100 Automated Microbiology System. Results were interpreted according to the Clinical and Laboratory Standards Institute (CLSI M100, 30th edition) breakpoints.

### Biofilm Formation Assay

Biofilm formation was estimated by crystal violet (CV) assay as previously described (Khajanchi et al., 2012; Fu et al., 2018). ATCC43816, Δ*qseB*, Δ*qseC*, Δ*qseBC*, ATCC43816-pBAD24, Δ*qseC*-pBAD24, and Δ*qseC*-pC*qseC* were grown overnight in LB broth and then sub-cultured 1:100 into fresh LB, and grown for 6 h; 20 μl of culture was transferred to a 96-cell polystyrene microtiter plate containing 180 μl brain heart infusion (BHI). Afterward, the plate was incubated at 37°C for 36 h. The medium was then decanted and planktonic cells were washed off gently with sterile water. Adhered biofilms were stained for 30 min at room temperature with 250 μl 1% CV (Beyotime, China), and the purple area was dissolved with 33% glacial acetic acid. Finally, biofilm quantification was measured by using microtiter plate reader (TECAN Infinite M200 Pro NanoQuant) at a wavelength of 595 nm. Absorbance data were obtained from three replicate experiments. In addition, we performed the above procedure by using plastic centrifuge tubes to visualize results of biofilm formation.

### Serum Killing Assay

Overnight cultures of ATCC43816, Δ*qseB*, Δ*qseC*, Δ*qseBC*, ATCC43816-pBAD24, Δ*qseC*-pBAD24, and Δ*qseC*-pC*qseC* grown in LB were sub-cultured 1:100 and grown to late exponential phase; 250 μl bacteria (10^5^CFU/ml) were incubated with 750 μl serum for 2 h. Samples were serially diluted and plated onto LB agar, and counted colonies were incubated overnight.

#### *Galleria mellonella* Killing Assay

*G. mellonella* larvae were purchased from Tianjin Huiyude Biotech Company, Tianjin, China. Each group included 40 larvae, and bacterial inoculum (10^5^CFU/larva) was administered as 10 μl injection into the left proleg. Saline inoculated larvae were included as negative control. Larvae were incubated at 37°C, in the dark, and scored every 24 h for survival or death (no response to stimulation recorded as dead). The killing assays were performed in triplicate using three different batches of *G. mellonella* larvae.

### RNA Sequencing and Differential Expression Analysis

The RNA-sequencing was performed by Shanghai Majorbio Bio-pharm Technology Co., Ltd. Total RNA was extracted from the late-exponentially growing ATCC43816, Δ*qseB*, Δ*qseC*, and Δ*qseBC* using TRIzol ^®^ Reagent (Invitrogen) followed by genomic DNA removal using DNase (Takara). RNAseq libraries were prepared according TruSeq™ RNA sample preparation Kit from Illumina (San Diego, CA, United States). The ribosomal RNA (rRNA) was removed using Ribo-Zero Magnetic Kit (epicenter). Paired-end RNA sequencing was performed with the Illumina HiSeq × TEN (2 × 150 bp read length).

The data were analyzed with the free online platform of Majorbio Cloud Platform.^2^ Raw sequencing reads were trimmed and filtered for high quality reads before being mapped to ATCC43816 genomes (Accession: PRJNA675363) using Bowtie2.^3^ RSEM^4^ was used to quantify expression levels of genes and transcripts. Differentially expressed genes (DEGs) were identified using DESeq2 packages^5^. The differential expression was considered as statistically significant if *p* < 0.05 and |log2(fold change)| > 2. KEGG and GO enrichment was conducted using KOBAS 2.0^6^ and Goatools.^7^

### Total RNA Isolation, cDNA Generation and Real-Time PCR Processing

Overnight culture of ATCC43816, Δ*qseB*, Δ*qseC*, Δ*qseBC*, ATCC43816-pBAD24, Δ*qseC*-pBAD24, and Δ*qseC*-pC*qseC* were diluted 1:100 in LB media and grown for 6 h to late exponential phase. Cells were collected and treated with Trizol (Sigma) to extract total RNA, followed by DNase treatment as above. The transcript levels of genes were tested by quantitative reverse-transcription PCR (qRT-PCR). HiScript III RT SuperMix kit and Taq Pro Universal SYBR qPCR Master Mix kit (Vazyme Biotech Co., Ltd., China) was used according to the manufacturer’s instructions. Differences in gene expression were normalized with the expression of 16S rRNA and calculated by the 2^–ΔΔCt^ method. All of the qRT-PCR assays were repeated at least three times.

### Statistical Analyses

The Student *t*-test was used to detect the differences between groups in the experiments. Survival curves were assessed by log-rank rest. *p* < 0.05 was considered statistically significant.

### Data Availability

The raw sequencing files in this study are available at the NCBI Sequence Read Archive (SRA) under BioProject PRJNA784457.

## Results

### Deletion of *qseB, qseC*, and *qseBC* Did Not Affect the Growth and Antimicrobial Susceptibility

**FIGURE 1:**
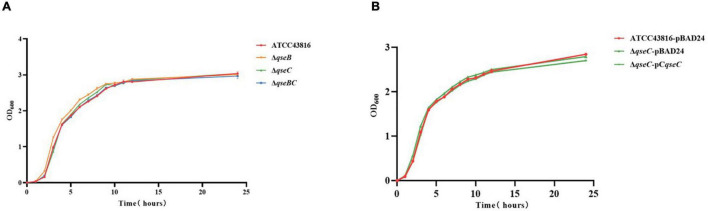
**(A)** The growth rates of ATCC43816, Δ*qseB*, Δ*qseC* and Δ*qseBC* were similar by measuring the optical density (OD_600_) at per hour over a period of 24 h. **(B)** Growth curves of ATCC43816-pBAD24, Δ*qseC*-pBAD24 and the complement strain Δ*qseC*-pCqseC.

To investigate the roles of QseBC in hvKP, Δ*qseB*, Δ*qseC*, and Δ*qseBC* were constructed by CRISPR/Cas9-mediated genome editing. The correct mutants were confirmed by PCR and Sanger sequencing. The colony morphologies of the three mutants on LB agar plates were similar to that of the wild-type. The growth curves of three mutants in LB broth over a period of 24 h were also similar to that of the wild-type strain (Figure 1). Antimicrobial susceptibility testing showed that ATCC43816, Δ*qseB*, Δ*qseC*, and Δ*qseBC* were susceptible to all tested antibacterial drugs. There was no significant difference of the minimum inhibitory concentration (MIC) values in ATCC43816, Δ*qseB*, Δ*qseC*, and Δ*qseBC* against 18 antibiotics examined (Supplementary Table 2). These results indicated that deletion of *qseB*, *qseC*, and *qseBC* did not affect growth and antimicrobial susceptibility of hvKP ATCC43816.

### Deletion of *qseC* Increased Biofilm Formation

**FIGURE 2:**
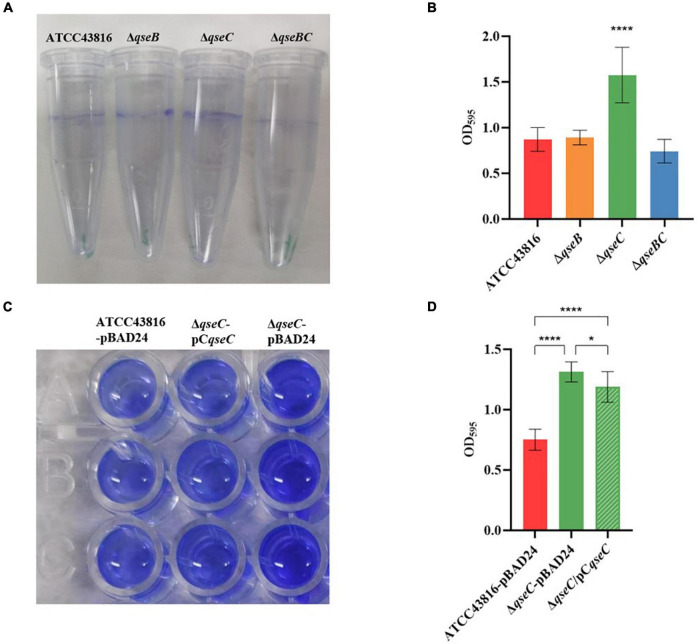
**(A)** Detection of biofilm formed by ATCC43816, Δ*qseB*, Δ*qseC* and Δ*qseBC*. Image of biofilm stained with 1% CV and adhered on plastic centrifuge tubes. **(B)** Biofilm were stained with 1% CV and washed with sterile water. The extracted color was dissolved with 33% glacial acetic acid and measured at OD_595_. Asterisks (****) represent statistically significant differences in biofilm formation between ATCC43816 and its *qseC*mutant (*P* < 0.0001, *t* test). **(C)** Image of biofilm formed by ATCC43816-pBAD24, Δ*qseC*-pBAD24 and the complement strain Δ*qseC*-pC*qseC*. Biofilm were stained with 1% CV, washed with sterile water and then dissolved with 33% glacial acetic acid. **(D)** The extracted color was measured at OD_595_ to show biofilm production. Asterisks (****) represent statistically significant differences in biofilm formation between ATCC43816-pBAD24 and Δ*qseC*-pBAD24/Δ*qseC*-pC*qseC* (*P* < 0.0001, *t* test). The biofilm formation of Δ*qseC*-pC*qseC* was significantly lower compared with that of Δ*qseC*-pBAD24 (*P* = 0.0366, *t* test).

**FIGURE 3:**
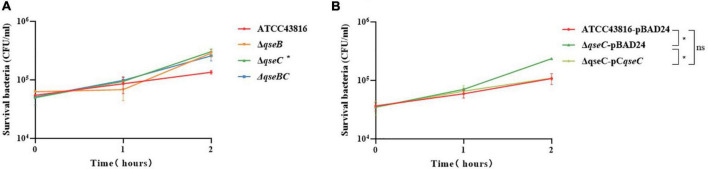
**(A)** Effect of human serum on ATCC43816, Δ*qseB*, Δ*qseC* and Δ*qseBC*. The bacteria were mixed with serum from healthy human volunteers for 2 hours. Δ*qseC* had significantly higher resistance when compared to control ATCC43816 (*P* = 0.0438, two tailed unpaired t test). **(B)** Effect of human serum on ATCC43816-pBAD24, Δ*qseC*-pBAD24 and the complement strain Δ*qseC*-pC*qseC*. Δ*qseC*-pBAD24 had significantly higher resistance when compared to control ATCC43816-pBAD24 (*P* = 0.0327, two tailed unpaired *t* test).

That QseBC regulated biofilm formation had been reported in *E. coli* (Sharma and Casey, 2014; Gou et al., 2019). Here, the CV staining assay was applied to examine whether QseBC affected biofilm formation in hvKP ATCC43816. As shown in Figure 2, Δ*qseC* formed a significantly increased, solid-surface-associated biofilm in the polystyrene plate, with about a twofold increase in the CV staining when compared with the biofilm formed by ATCC43816. Compensation of *qseC* partially decreased the biofilm formation (Figures 3B,C). Since growth was not altered after *qseC*-deletion (Figure 1), the biofilm changes were not a result of growth differences. However, Δ*qseB* and Δ*qseBC* produced a similar level of biofilms to that from the wild-type strain. These data suggested to us that QseC negatively controlled biofilm formation in hvKP ATCC43816.

### Deletion of *qseC* Increased Serum Resistance of ATCC43816

Serum killing assay was used to study serum resistance in the wild-type and mutants *in vitro.* Viable counts of both wild-type strain and mutants were > 100% throughout the time period, suggesting that they all had a high level of defense against serum bactericidal activity (Figure 3A). A significant difference was identified between ATCC43816 and Δ*qseC*. After 2 h of incubation, the mean survival count of Δ*qseC* showed 3.02 × 10^5^ CFU/ml and exhibited ∼3 times higher than that of ATCC43816 and complemented strain Δ*qseC*-pC*qseC* (Figure 3B), while serum-resistant levels of Δ*qseB* and Δ*qseBC* had no significant difference with that of ATCC43816 in serum killing assay.

### Deletion of *qseC* Increased *in vivo* Virulence of ATCC43816

**FIGURE 4:**
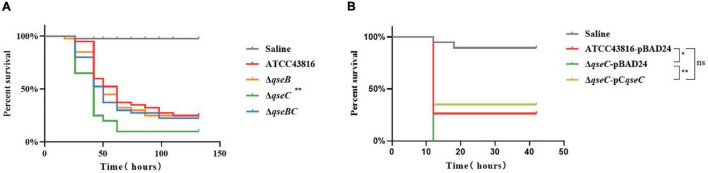
**(A)** Larvae survival of ATCC43816, Δ*qseB*, Δ*qseC* and Δ*qseBC*. *Galleria mellonella* were injected with 105 CFU wild-type and mutants (40 larvae per group). The survival of *G. mellonella* infected with Δ*qseC* was significantly lower compared with that of the wild-type group (*P* = 0.0012, Log-rank test). **(B)** Larvae survival of ATCC43816-pBAD24, Δ*qseC*-pBAD24 and the complement strain Δ*qseC*-pC*qseC*. The survival of *G. mellonella* infected with Δ*qseC*-pC*qseC* was significantly higher than that of Δ*qseC*-pBAD24 (*P* = 0.0040, Log-rank test).

The *Galleria mellonella* infection model has been suggested to be a valid method for evaluating *K. pneumoniae* pathogenicity (Insua et al., 2013). To examine the influence of QseBC on *in vivo* virulence of hvKP ATCC43816, the *G. mellonella* killing assay was then performed. The *qseB* and *qseBC* mutants remained as viable as ATCC43816 in experimentally infected *G. mellonella*. After 72 h of infection, the survival rate of Δ*qseC* was about less than 20%. By contrast, the survival rate of ATCC43816, *qseB*, and *qseBC* mutants was about 40%, which was significantly higher than that of the Δ*qseC* (Figure 4A). As shown in Figure 4B, the survival rate was significantly increased in Δ*qseC*-pC*qseC* relative to Δ*qseC*. These data demonstrated that Δ*qseC* had relatively higher *in vivo* virulence in comparison to the wild-type strain.

### Profiling Gene Expression of Δ*qseB*, Δ*qseC*, and Δ*qseBC*

**FIGURE 5:**
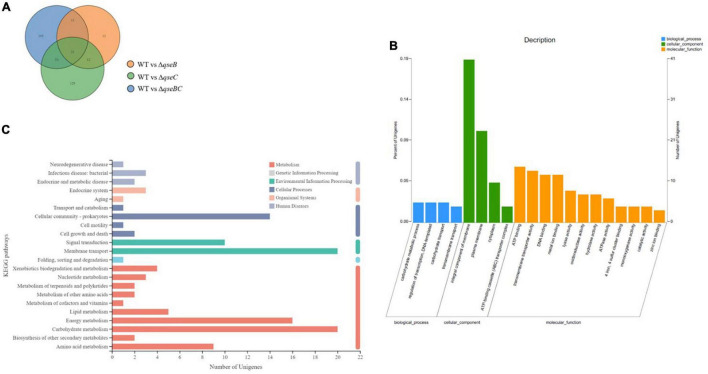
Analysis of total RNA sequencing. **(A)** The Venn diagram shows the overlapped DEGs numbers across Δ*qseB*, Δ*qseC* and Δ*qseBC*. **(B)** Significantly enriched GO terms of DGEs in the *qseC*mutant. **(C)** Significantly enriched KEGG pathways of DEGs in the *qseC*mutant.

**FIGURE 6:**
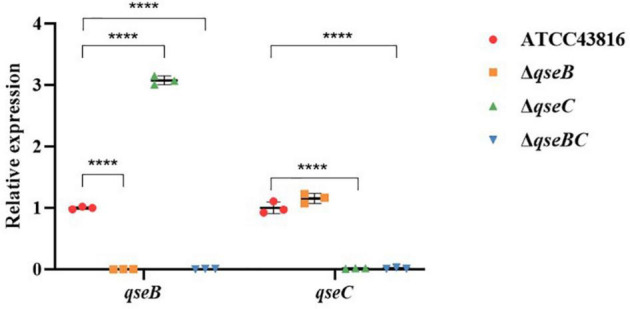
Expression of genes *qseB*, *qseC* in ATCC43816, Δ*qseB*, Δ*qseC* and Δ*qseBC*. Cultures of the wild-type and mutants were grown in LB medium to late-exponential phase, and expressions of genes *qseC*, *qseB* were measured by qRT-PCR and normalized to the expression in the wild-type strain. Data were mean ± standard deviation of three replicates. Significant expression differences between the wild-type andmutants were determined using two-way ANOVA, ****P < 0.0001.

**FIGURE 7:**
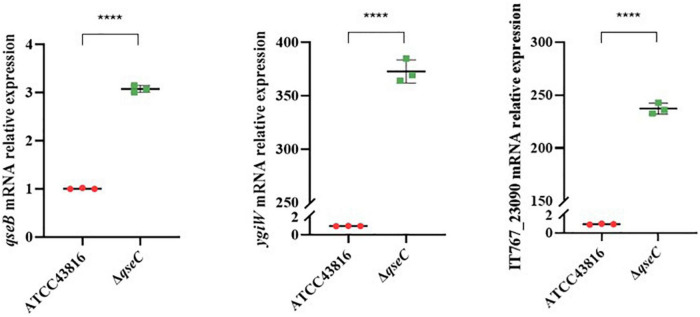
**(A)** Expression of genes *qseB*, *ygiW*, IT767_23090 in ATCC43816 and Δ*qseC*. Cultures of ATCC43816 and Δ*qseC* were grown in LB medium to late-exponential phase, and expressions of genes *qseB*, *ygiW*, IT767_23090 were measured by qRT-PCR and normalized to the expression in the wild-type strain. Data were mean ± standard deviation of three replicates. Significant expression differences between the wild-type and *qseC*mutant were determined using *t* test. ****P < 0.0001. **(B)** Expression of genes *qseB*, *ygiW*, IT767_23090 in ATCC43816-pBAD24, Δ*qseC*-pBAD24 and the complement strain Δ*qseC*-pC*qseC*. Data were mean ± standard deviation of three replicates. Significant expression differences between the wild-type and *qseC*mutant were determined using *t* test. *P < 0.05, ***P < 0.001, ****P < 0.0001.

To explore possible transcriptomic contributions of QseBC to virulence in hvKP ATCC43816, we examined the gene expression profiles of the late-exponentially growing ATCC43816, Δ*qseB*, Δ*qseC*, and Δ*qseBC*. We identified differentially expressed genes (DEGs) by comparing the RNA-sequencing data with the control strain ATCC43816. DEGs were identified by adjusted *p* < 0.05 and |log2(fold change)| > 2. Compared to the wild-type strain, the number of DEGs in Δ*qseB*, Δ*qseC*, and Δ*qseBC* groups was 58 (49 upregulated and 9 downregulated genes), 216 (186 upregulated and 30 downregulated genes), and 193 (171 upregulated and 22 downregulated genes), respectively (Supplementary Tables 3–5). In our analysis, we detected 12 DEGs that were shared by Δ*qseB* and Δ*qseC*, 13 DEGs shared by Δ*qseB* and Δ*qseBC*, 54 DEGs shared by Δ*qseC* and Δ*qseBC*, and 21 DEGs shared by all three mutants (Figure 5A). Notably, among the 129 DEGs unique to Δ*qseC*, IT767_23090 (AraC family transcriptional regulator family) and *ygiW* showed the highest expression levels (up to 300-fold increase). Compared with ATCC43816, Δ*qseC* apparently had a significantly high-expression of the *qseB* gene. The gene expressions of selected genes were confirmed by qRT-PCR and the qRT-PCR showed similar results as the RNA-seq analysis (Figures 6, 7A). The transcript levels of *qseB*, *ygiW*, and *araC* were restored in Δ*qseC*-pC*qseC* (Figure 7B).

The 216 DEGs of Δ*qseC* group were performed by Gene Ontology (GO) enrichment analysis. Twenty significantly enriched GO terms from three categories (biological process, cellular component, and molecular function) were shown in Figure 5B. Most DEGs were enriched in two GO terms of cellular component, integral component of membrane and plasma membrane. In addition, DGEs were also subject to KEGG pathway enrichment analysis. Cellular community, signal transduction, membrane transport, energy metabolism, carbohydrate metabolism, and amino acid metabolism were significantly enriched in KEGG pathways (Figure 5C). Among the cellular community pathway, genes associated biofilm formation (*glgC, glgP, glgA, gcvA, bcsA, ydaM, paaF, ptsG*) in Δ*qseC* were significantly up-regulated as comparison with the control, which is consistent with the increased biofilm production described above. Moreover, several up-regulated DEGs in pathway involved in virulence, including the bacterial secretion system (*virB4, virB6, virB10, vgrG, hcp*) and biosynthesis of the siderophore group (*entC, entD, entE*), were identified. The upregulation of these genes may be associated with the increased virulence observed in the *in vivo* and *in vitro* models, which deserve further studies.

## Discussion

In this study, we investigated the effect of QseBC on the pathogenic bacteria virulence and biofilm formation in hvKp strain ATCC43816. The results showed that *qseC*-deletion increased serum resistance in the serum killing assay, but Δ*qseB* and Δ*qseBC* showed no significant difference compared with the wild-type strain. The results suggested that Δ*qseC* could increase serum resistance and possibly enhances the virulence of hvKP. In order to further test this hypothesis, we conducted the *in vivo G. mellonella* killing assay. The results showed that there was a significantly higher virulence of the *qseC* mutant strain when compared to the control ATCC43816. The results of biofilm formation testing also showed that there was a significantly increased biofilm production in Δ*qseC*, while the levels of biofilm production in Δ*qseB* and Δ*qseBC* mutants were similar to those from the wild-type strain. These data suggested that the QseC negatively controlled biofilm formation in hvKP ATCC43816, and Δ*qseC*, but not Δ*qseB* and Δ*qseBC*, had higher serum resistance and *in vivo* virulence in the *G. mellonella* model. Several previous studies have demonstrated that QseC has a regulatory function on the motility, biofilm formation, and virulence in some other pathogens. For example, Yang et al. (2014) found that motility was diminished and biofilm formation was decreased in an *E. coli qseC* mutant. However, our study found that QseC is negatively correlated with the virulence in ATCC43816. Our result appears to be contradicted with the results for *E. coli*, and an explanation is the TCS QseBC system may behave differently among different species.

In order to further explore the mechanism how QseC negatively regulate the virulence, transcriptional profiling was implemented. The RNA-seq results suggested that in Δ*qseC*, the gene associated with biofilm formation (*glgC, glgP, glgA, gcvA, bcsA, ydaM, paaF, ptsG*), bacterial type VI secretion system (*virB4, virB6, virB10, vgrG, hcp*), and biosynthesis of siderophore (*entC, entD, entE*) were significantly up-regulated in comparison to the control. It is worth noting that deletion of *qseC* resulted in the highest overexpression of *ygiW* and *qseB*. In the oral pathogen *Aggregatibacter actinomycetemcomitans*, the *qseBC* genes are co-expressed with *ygiW*, and the transcription of *ygiW-qseBC* operon is directed by the promoter in upstream region of *ygiW* that contains QseB binding sites (Juárez-Rodríguez et al., 2013, 2014). However, analysis of the ATCC43816 genome (accession no. CP009208) showed that *ygiW* was transcribed from the opposite strand as *qseBC* (Weigel and Demuth, 2016), which also suggested that the gene expression regulation for *qseBC* may be different in *A. actinomycetemcomitans* and *K. pneumoniae*. In the absence of the sensor protein QseC, QseB regulator cannot receive the signal of automatic phosphorylation. Therefore, the expression of *qseB* is supposed to be downregulated due to the lack of autophosphorylation signals from QseC. However, in our study, the expression of *qseB* was significantly upregulated by approximately four times, indicating that QseB could accept autophosphorylation signals from other sources. For example, Guckes et al. (2013) reported that in uropathogenic *E. coli*, polymyxin resistance (Pmr) sensor kinase PmrB could active QseB in the absence of QseC. Interestingly, Δ*qseB* and Δ*qseBC* have similar levels of biofilm formation, serum resistance, and *G. mellonella* killing effects to those from the wild-type strain. We therefore hypothesized that in the presence of both QseB and QseC, QseC works as sensor and receives signal, and transfers the phosphate groups to the intracellular response regulator QseB. The phosphorylated QseB directly regulates the expression of iron carrier-related genes, and other virulence-related genes, leading to the changes of bacterial virulence and biofilm formation. In the absence of QseC, QseB could compensatorily receive phosphate groups from other sensor kinase, while the compensatory pathway may be inhibited in the normal condition. When *qseB* was knocked out (both in Δ*qseB* and Δ*qseBC*), the *qseB* regulatory pathway was interrupted, and Δ*qseB* and Δ*qseBC* behaved similarly in the *in vivo* and *in vitro* models.

We also analyzed the differential gene expressions between Δ*qseB*, Δ*qseC*, Δ*qseBC*. IT767_23090, belonging to AraC family transcriptional regulator family, was upregulated by 305-fold. In *Bacillus subtilis*, the AraC family transcriptional regulatory protein PrkC is used to phosphorylate proteins in the metabolic pathway (Libby et al., 2015). In *Vibrio cholerae*, AraC family transcribed regulated protein ToxT promotes virulence by activating cholera toxin and coordinately regulating the expression of fimbriae (Thomson et al., 2015). The AraC family transcribed regulatory protein RarA leads to multiple drug resistance by regulating the expression of active efflux aggregation in *K. pneumoniae* (Jiménez-Castellanos et al., 2016). The study by Chen et al. (2015) showed that the AraC family transcription regulator protein RSP increases the expression of *Staphylococcus aureus* virulence by upregulating the accessory gene regulator (Agr) controlled toxins phenol-soluble modules (PSMs) and alpha-toxin. As such, we suspected that Δ*qseC* may in part work through upregulating the AraC family transcriptional regulator IT767_23090 to confer to higher virulence in hvKP ATCC43816. However, further studies are needed to determine the role of IT767_23090 in the increased virulence in Δ*qseC.*

In summary, our data showed that Δ*qseC* leads to the high expression of *qseB, ygiW*, IT767_23090, and genes associated biofilm formation, bacterial type VI secretion system, and biosynthesis of siderophore, which might contribute to the increased of virulence and biofilm formation. This research provides new insights into the role of QseBC in biofilm formation and virulence of hvKP ATCC43816.

## Data Availability Statement

The datasets presented in this study can be found in online repositories. The names of the repository/repositories and accession number(s) can be found in the article/Supplementary Material.

## Author Contributions

JL, JZ, and TW contributed equally to this work. The order of co-first authors was determined by discussion and mutual agreement between three co-first authors. HD and FZ are corresponding authors. All authors contributed to the article and approved the submitted version.

## Conflict of Interest

The authors declare that the research was conducted in the absence of any commercial or financial relationships that could be construed as a potential conflict of interest.

## Publisher’s Note

All claims expressed in this article are solely those of the authors and do not necessarily represent those of their affiliated organizations, or those of the publisher, the editors and the reviewers. Any product that may be evaluated in this article, or claim that may be made by its manufacturer, is not guaranteed or endorsed by the publisher.

## Footnotes

1
http://crispr.dfci.harvard.edu/SSC/

2
www.majorbio.com

3
http://bowtie-bio.sourceforge.net/bowtie2/index.shtml

4
http://deweylab.github.io/RSEM/

5
https://genomebiology.biomedcentral.com/articles/10.1186/s13059-014-0550-8

6
http://kobas.cbi.pku.edu.cn

7
https://github.com/tanghaibao/GOatools

## References

[B1] Abisado R. G. Benomar S. Klaus J. R. Dandekar A. A. Chandler J. R. (2018). Bacterial quorum sensing and microbial community interactions. *mBio* 9:e02331-17. 10.1128/mBio.02331-17 PMC5964356 29789364

[B2] Bearson B. L. Bearson S. M. (2008). The role of the QseC quorum-sensing sensor kinase in colonization and norepinephrine-enhanced motility of *Salmonella enterica* serovar Typhimurium. *Microb. Pathog.* 44 271–278. 10.1016/j.micpath.2007.10.001 17997077

[B3] Chen Z. Wang Y. Tian L. Zhu X. Li L. Zhang B. (2015). First report in China of *Enterobacteriaceae* clinical isolates coharboring blaNDM-1 and blaIMP-4 drug resistance genes. *Microb. Drug Resist.* 21 167–170. 10.1089/mdr.2014.0087 25389598

[B4] Choby J. E. Howard-Anderson J. Weiss D. S. (2020). Hypervirulent *Klebsiella pneumoniae* - clinical and molecular perspectives. *J. Intern. Med.* 287 283–300. 31677303 10.1111/joim.13007 PMC7057273

[B5] Clarke M. B. Hughes D. T. Zhu C. Boedeker E. C. Sperandio V. (2006). The QseC sensor kinase: a bacterial adrenergic receptor. *Proc. Natl. Acad. Sci. U. S. A.* 103 10420– 10425. 16803956 10.1073/pnas.0604343103 PMC1482837

[B6] Fu L. Huang M. Zhang X. Yang X. Liu Y. Zhang L. (2018). Frequency of virulence factors in high biofilm formation bla(KPC-2) producing *Klebsiella pneumoniae* strains from hospitals. *Microb. Pathog.* 116 168–172. 10.1016/j.micpath.2018.01.030 29360567

[B7] Gou Y. Liu W. Wang J. J. Tan L. Hong B. Guo L. (2019). CRISPR-Cas9 knockout of qseB induced asynchrony between motility and biofilm formation in *Escherichia coli*. *Can. J. Microbiol.* 65 691–702. 10.1139/cjm-2019-0100 31075206

[B8] Groisman E. A. (2016). Feedback control of two-component regulatory systems. *Annu. Rev. Microbiol.* 70 103–124. 10.1146/annurev-micro-102215-095331 27607549 PMC8380452

[B9] Guckes K. R. Kostakioti M. Breland E. J. Gu A. P. Shaffer C. L. Martinez C. R. III (2013). Strong cross-system interactions drive the activation of the QseB response regulator in the absence of its cognate sensor. *Proc. Natl. Acad. Sci. U. S. A.* 110 16592–16597. 10.1073/pnas.1315320110 24062463 PMC3799328

[B10] Hao M. He Y. Zhang H. Liao X. P. Liu Y. H. Sun J. (2020). crispr-cas9-mediated carbapenemase gene and plasmid curing in carbapenem-resistant *Enterobacteriaceae*. *Antimicrob. Agents Chemother.* 64:e00843-20. 10.1128/AAC.00843-20 32631827 PMC7449206

[B11] Harada S. Aoki K. Yamamoto S. Ishii Y. Sekiya N. Kurai H. (2019). Clinical and molecular characteristics of *Klebsiella pneumoniae* isolates causing bloodstream infections in Japan: occurrence of hypervirulent infections in health care. *J. Clin. Microbiol.* 57:e01206-19. 10.1128/JCM.01206-19 31434721 PMC6812994

[B12] Insua J. L. Llobet E. Moranta D. Pérez-Gutiérrez C. Tomás A. Garmendia J. (2013). Modeling *Klebsiella pneumoniae* pathogenesis by infection of the wax moth Galleria mellonella. *Infect. Immun.* 81 3552–3565. 10.1128/IAI.00391-13 23836821 PMC3811777

[B13] Jiménez-Castellanos J. C. Wan Ahmad Kamil W. N. Cheung C. H. Tobin M. S. Brown J. Isaac S. G. (2016). Comparative effects of overproducing the AraC-type transcriptional regulators MarA, SoxS, RarA and RamA on antimicrobial drug susceptibility in *Klebsiella pneumoniae*. *J. Antimicrob. Chemother.* 71 1820–1825. 10.1093/jac/dkw088 27029850 PMC4896410

[B14] Juárez-Rodríguez M. D. Torres-Escobar A. Demuth D. R. (2013). ygiW and qseBC are co-expressed in Aggregatibacter actinomycetemcomitans and regulate biofilm growth. *Microbiology* 159 989–1001. 10.1099/mic.0.066183-0 23519160 PMC3709689

[B15] Juárez-Rodríguez M. D. Torres-Escobar A. Demuth D. R. (2014). Transcriptional regulation of the Aggregatibacter actinomycetemcomitans ygiW-qseBC operon by QseB and integration host factor proteins. *Microbiology* 160 2583–2594. 10.1099/mic.0.083501-0 25223341 PMC4252909

[B16] Khajanchi B. K. Kozlova E. V. Sha J. Popov V. L. Chopra A. K. (2012). The two-component QseBC signalling system regulates in vitro and in vivo virulence of Aeromonas hydrophila. *Microbiology* 158 259–271. 10.1099/mic.0.051805-0 21998161 PMC3352359

[B17] Libby E. A. Goss L. A. Dworkin J. (2015). The Eukaryotic-like Ser/Thr Kinase PrkC regulates the essential WalRK two-component system in *Bacillus subtilis*. *PLoS Genet.* 11:e1005275. 10.1371/journal.pgen.1005275 PMC4478028 26102633

[B18] Merighi M. Septer A. N. Carroll-Portillo A. Bhatiya A. Porwollik S. Mcclelland M. (2009). Genome-wide analysis of the PreA/PreB (QseB/QseC) regulon of *Salmonella enterica* serovar Typhimurium. *BMC Microbiol.* 9:42. 10.1186/1471-2180-9-42 PMC2653508 19236707

[B19] Novak E. A. Shao H. Daep C. A. Demuth D. R. (2010). Autoinducer-2 and QseC control biofilm formation and in vivo virulence of Aggregatibacter actinomycetemcomitans. *Infect Immun* 78 2919–2926. 10.1128/IAI.01376-09 20404080 PMC2897384

[B20] Russo T. A. Marr C. M. (2019). Hypervirulent *Klebsiella pneumoniae*. *Clin. Microbiol. Rev.* 32:e00001-19. 10.1128/CMR.00001-19 PMC6589860 31092506

[B21] Sharma V. K. Casey T. A. (2014). *Escherichia coli* O157:H7 lacking the qseBC-encoded quorum-sensing system outcompetes the parental strain in colonization of cattle intestines. *Appl. Environ. Microbiol.* 80 1882–1892. 10.1128/AEM.03198-13 24413602 PMC3957642

[B22] Sperandio V. Torres A. G. Kaper J. B. (2002). Quorum sensing *Escherichia coli* regulators B and C (QseBC): a novel two-component regulatory system involved in the regulation of flagella and motility by quorum sensing in *E. coli*. *Mol. Microbiol.* 43 809–821. 10.1046/j.1365-2958.2002.02803.x 11929534

[B23] Thomson J. J. Plecha S. C. Withey J. H. (2015). A small unstructured region in *Vibrio cholerae* ToxT mediates the response to positive and negative effectors and ToxT proteolysis. *J. Bacteriol.* 197 654–668. 10.1128/JB.02068-14 25422303 PMC4285994

[B24] Wang X. Wang Q. Yang M. Xiao J. Liu Q. Wu H. (2011). QseBC controls flagellar motility, fimbrial hemagglutination and intracellular virulence in fish pathogen Edwardsiella tarda. *Fish Shellfish Immunol.* 30 944–953. 10.1016/j.fsi.2011.01.019 21288493

[B25] Wang Y. Wang S. Chen W. Song L. Zhang Y. Shen Z. (2018). CRISPR-Cas9 and CRISPR-assisted cytidine deaminase enable precise and efficient genome editing in *Klebsiella pneumoniae*. *Appl. Environ. Microbiol.* 84:e01834-18. 10.1128/AEM.01834-18 30217854 PMC6238054

[B26] Weigel W. A. Demuth D. R. (2016). QseBC, a two-component bacterial adrenergic receptor and global regulator of virulence in *Enterobacteriaceae* and Pasteurellaceae. *Mol. Oral Microbiol.* 31 379–397. 10.1111/omi.12138 26426681 PMC5053249

[B27] Yang K. Meng J. Huang Y. C. Ye L. H. Li G. J. Huang J. (2014). The role of the QseC quorum-sensing sensor kinase in epinephrine-enhanced motility and biofilm formation by *Escherichia coli*. *Cell Biochem. Biophys.* 70, 391–398. 10.1007/s12013-014-9924-5 24676679

[B28] Zafar S. Hanif S. Akhtar H. Faryal R. (2019). Emergence of hypervirulent K. pneumoniae causing complicated UTI in kidney stone patients. *Microb. Pathog.* 135:103647. 10.1016/j.micpath.2019.103647 31356929

[B29] Zhu J. Wang T. Chen L. Du H. (2021). Virulence factors in hypervirulent *Klebsiella pneumoniae*. *Front. Microbiol.* 12:642484. 10.3389/fmicb.2021.642484 PMC8060575 33897652

