# Supplementary material for: The Role of the Two-Component QseBC Signaling System in Biofilm Formation and Virulence of Hypervirulent Klebsiella pneumoniae ATCC43816
Source: Front Microbiol. 2022 Apr 6;13:817494. doi: 10.3389/fmicb.2022.817494 (PMC9019566; doi:10.3389/fmicb.2022.817494)
Supplement: Supplementary file 1 [file Table_1.docx]

| **Table S1 Strains, plasmids and primers used in this study** | | |
| --- | --- | --- |
| **Strains/Plasmids/Primers** | **Description/Nucleotide sequence (5’→ 3’)** | **Source/reference/function** |
| **Strains** |  |  |
| *E. coli* DH5α |  | Laboratory stock |
| *K. pneumoniae* ATCC43816 | Wild type | American Type Culture Collection |
| ATCC43816Δ*qseB* | ATCC43816 *qseB*-deletion mutant | This study |
| ATCC43816Δ*qseC* | ATCC43816 *qseC*-deletion mutant | This study |
| ATCC43816Δ*qseBC* | ATCC43816 *qseBC*-deletion mutant | This study |
| ATCC43816-pBAD24 | ATCC43816 with the empty vector pBAD24, Ampicillin resistance | This study |
| ATCC43816Δ*qseC*-pBAD24 | ATCC43816Δ*qseC* with the empty vector pBAD24, Ampicillin resistance | This study |
| ATCC43816Δ*qseC*-pC*qseC* | ATCC43816Δ*qseC* with the complement plasmid pC*qseC*, Ampicillin resistance | This study |
| **Plasmids** |  |  |
| pCasKP | Temperature sensitive, apramycin resistance | (Hao et al., 2020) |
| pSGKP | Rifampicin resistance | (Hao et al., 2020) |
| pBAD24 | L-arabinose-inducible expression plasmid | Laboratory stock |
| **Primers** |  |  |
| HindⅢ-QseB-N20-For | cccAAGCTTTGCTCTGGCCGTGCGCGCGGGTTTTAGAGCTAGAAATAGCAAGTT | Construct pSGKP with targeted sgRNA (pSGKP-QseB/QseC-N20) |
| HindⅢ-QseC-N20-For | cccAAGCTTGCTGGCCATCCCCCAGGCGGGTTTTAGAGCTAGAAATAGCAAGTT |  |
| XbaⅠ-gRNA-Rev | gcTCTAGACCGGGCTGCAGGAATT |  |
| M13-Rev | CAGGAAACAGCTATGACC |  |
| QseB-1 | CACGATGAGCAGCACGAACATCA | Construct a homologous recombination arm as the *qseB*-deletion repair template |
| QseB-2 | GAGGGAAACAAAACAACTTGCCCGCCTGAGC |  |
| QseB-3 | CAAGTTGTTTTGTTTCCCTCCGACGATCCCGT |  |
| QseB-4 | CGACGGTCACGCCGTTCCA |  |
| QseC-1 | TGGAAGAACTGGTCCTGGTCG | Construct a homologous recombination arm as the *qseC*-deletion repair template |
| QseC-2 | TGGGTGACGCCGAGATTATTGCTCATTTGGCAA |  |
| QseC-3 | AATAATCTCGGCGTCACCCAGGGTATAGC |  |
| QseC-4 | ATGCGCATATTACTGGTGGAAGAT |  |
| QseBC-1 | TGGAAGAACTGGTCCTGGTC | Construct a homologous recombination arm as the *qseBC*-deletion repair template |
| QseBC-2 | GAGGGAAACCGAGATTATTGCTCATTTGGCA |  |
| QseBC-3 | GAGCAATAATCTCGTTATGGCGCGATTTTACGG |  |
| QseBC-4 | TTATGGCGCGATTTTACGG |  |
| QseBC-Up | TGCCAAATGAGCAATAATCTCG | Confirm the correct Δ*qseB/*Δ*qsec/*Δ*qseBC* mutants |
| QseBC-Down | GGATCGTCGGAGGGAAAC |  |
| QseB-For | ATAAAGTCGCTGCCGAGCTT |  |
| QseB-Rev | TGAGCCGGTGCTGATCCTCA |  |
| QseC-For | CTGCTGCACATCATCCAGTT |  |
| QseC-Rev | CTGATGTTCGTGCTGCTCAT |  |
| QseC-C-For | TCATCCGCCACTACCAGTACACGCGGGCC | Construct and confirm the recombinant plasmid pBAD24-*qseC* |
| QseC-C-Rev | GTTTTTTTGGATGAAACAACTTGCCCGCC |  |
| pBAD24-C-For | GTACTGGTAGTGGCGGATGAGAGAAGATT |  |
| pBAD24-C-Rev | GTTGTTTCATCCAAAAAAACGGGTATGGAGA |  |
| pBAD24-For | ACATTGATTATTTGCACGGCGT |  |
| pBAD24-Rev | CAGACCGCTTCTGCGTTCTG |  |
| qRT-QseB-For | GTCAAGGATTGCGGCGTCGTAG | Primers used for quantitative reverse-transcription PCR (qRT-PCR) |
| qRT-QseB-Rev | GCATATTACTGGTGGAAGATGACAAGC |  |
| qRT-QseC-For | CGACGGCGATGCGGTAGC |  |
| qRT-QseC-Rev | ACGGCGAGAACGGAGAGGAT |  |
| qRT-YgiW-For | CGATGACCGCGATTATTGCTTTGG |  |
| qRT-YgiW-Rev | GCCTGGGTGGTAGTGGTAGTCG |  |
| qRT-AraC-For | GCCATCTTGATGAGCCGCTGTC |  |
| qRT-AraC-Rev | TCGCAGACGCAACCACTGAATATAC |  |
