# Supplementary material for: The Role of the Two-Component QseBC Signaling System in Biofilm Formation and Virulence of Hypervirulent Klebsiella pneumoniae ATCC43816
Source: Front Microbiol. 2022 Apr 6;13:817494. doi: 10.3389/fmicb.2022.817494 (PMC9019566; doi:10.3389/fmicb.2022.817494)
Supplement: Supplementary file 2 [file Table_2.docx]

| **Table S2. Antimicrobial susceptibility profile for ATCC43816, Δ*qseB*, Δ*qseC,* Δ*qseBC*,** **ATCC43816-pBAD24, Δ*qseC* -pBAD24 and Δ*qseC*-pCqseC.** | | | | | | | |
| --- | --- | --- | --- | --- | --- | --- | --- |
| **Antimicrobials^a^** | **ATCC43816** | **ATCC43816/Δ*qseB*** | **ATCC43816/Δ*qseC*** | **ATCC43816/Δ*qseBC*** | **ATCC43816-pBAD24** | **Δ*qseC* -pBAD24** | **Δ*qseC*-pCqseC** |
| AMK | <=8 | <=8 | <=8 | <=8 | <=8 | <=8 | <=8 |
| ATM | <=2 | <=2 | <=2 | <=2 | <=2 | <=2 | <=2 |
| CZ | <=2 | <=2 | <=2 | <=2 | <=2 | <=2 | <=2 |
| FOX | <=4 | <=4 | <=4 | <=4 | <=4 | <=4 | <=4 |
| CAZ | <=1 | <=1 | <=1 | <=1 | <=1 | <=1 | <=1 |
| CRO | <=1 | <=1 | <=1 | <=1 | <=1 | <=1 | <=1 |
| C | <=4 | <=4 | <=4 | <=4 | <=4 | <=4 | <=4 |
| CIP | <=0.5 | <=0.5 | <=0.5 | <=0.5 | <=0.5 | <=0.5 | <=0.5 |
| COL | <=1 | <=1 | <=1 | <=1 | <=1 | <=1 | <=1 |
| ETP | <=0.25 | <=0.25 | <=0.25 | <=0.25 | <=0.25 | <=0.25 | <=0.25 |
| FOS | <=16 | <=16 | <=16 | <=16 | <=16 | <=16 | <=16 |
| GM | <=2 | <=2 | <=2 | <=2 | <=2 | <=2 | <=2 |
| IPM | 0.5 | 0.5 | 0.5 | 0.5 | 0.5 | 0.5 | 0.5 |
| MEM | <=0.125 | <=0.125 | <=0.125 | <=0.125 | <=0.125 | <=0.125 | <=0.125 |
| MXF | <=0.5 | <=0.5 | <=0.5 | <=0.5 | <=0.5 | <=0.5 | <=0.5 |
| TET | <=2 | <=2 | <=2 | <=2 | <=2 | <=2 | <=2 |
| TIG | <=1 | <=1 | <=1 | <=1 | <=1 | <=1 | <=1 |
| TOB | <=2 | <=2 | <=2 | <=2 | <=2 | <=2 | <=2 |

^a^ Abbreviations: AMK, amikacin; ATM, aztreonam; CZ, cefazolin; FOX, cefoxitin; CAZ, ceftazidime; CRO, ceftriaxone; C, chloramphenicol; CIP, ciprofloxacin; COL, colistin; ETP, ertapenem; FOS, fosfomycin; GM, gentamycin; IPM, imipenem; MEM, meropenem; MXF, moxifloxacin; TET, tetracycline; TIG, tigecycline; TOB, tobramycin.
