# Supplementary material for: The Role of the Two-Component QseBC Signaling System in Biofilm Formation and Virulence of Hypervirulent Klebsiella pneumoniae ATCC43816
Source: Front Microbiol. 2022 Apr 6;13:817494. doi: 10.3389/fmicb.2022.817494 (PMC9019566; doi:10.3389/fmicb.2022.817494)
Supplement: Supplementary file 3 [file Table_3.docx]

| **Table S3 Differentially expressed genes in Δ*qseB* relative to the wild-type strain ATCC43816** | | | |
| --- | --- | --- | --- |
| Gene_id | Gene name | Gene description | Log_2_FC |
| sRNA0291 |  |  | 6.103202 |
| sRNA0302 |  |  | 6.103202 |
| IT767_16840 | IT767_16840 | phosphodiesterase | 3.472009 |
| IT767_14425 | IT767_14425 | fimbria/pilus periplasmic chaperone | 3.231 |
| sRNA0407 |  |  | 2.521591 |
| IT767_11570 | IT767_11570 | ABC transporter permease | 2.517444 |
| IT767_14540 | gabP | GABA permease | 2.485265 |
| IT767_11790 | IT767_11790 | two-component-system connector protein YcgZ | 2.449641 |
| IT767_09605 | IT767_09605 | general stress protein | 2.433153 |
| IT767_06320 | IT767_06320 | ferritin-like domain-containing protein | 2.420662 |
| IT767_14360 | phoE | phosphoporin PhoE | 2.38445 |
| IT767_06280 | IT767_06280 | hypothetical protein | 2.367179 |
| IT767_24725 | ssuD | FMNH2-dependent alkanesulfonate monooxygenase | 2.356531 |
| IT767_21320 | glpD | glycerol-3-phosphate dehydrogenase | 2.336849 |
| IT767_08665 | IT767_08665 | DUF3131 domain-containing protein | 2.310193 |
| sRNA0083 |  |  | 2.287819 |
| IT767_00735 | eutB | ethanolamine ammonia-lyase subunit alpha | 2.261283 |
| IT767_09600 | IT767_09600 | ferritin-like domain-containing protein | 2.242875 |
| IT767_14295 | IT767_14295 | CS1-pili formation C-terminal domain-containing protein | 2.226763 |
| IT767_21305 | glgP | glycogen phosphorylase | 2.226463 |
| IT767_06325 | IT767_06325 | hypothetical protein | 2.216354 |
| IT767_10170 | IT767_10170 | hypothetical protein | 2.212141 |
| IT767_18135 | IT767_18135 | CsbD family protein | 2.196435 |
| sRNA0218 |  |  | 2.192625 |
| sRNA0275 |  |  | 2.192625 |
| sRNA0246 |  |  | 2.192625 |
| sRNA0003 |  |  | 2.192625 |
| sRNA0356 |  |  | 2.192625 |
| IT767_06315 | IT767_06315 | hypothetical protein | 2.182611 |
| IT767_21300 | glgA | glycogen synthase GlgA | 2.175166 |
| IT767_22740 | lsrG | (4S)-4-hydroxy-5-phosphonooxypentane-2%2C3-dione isomerase | 2.168459 |
| IT767_00750 | eutK | ethanolamine utilization microcompartment protein EutK | 2.156233 |
| sRNA0118 |  |  | 2.140803 |
| sRNA0213 |  |  | 2.114301 |
| IT767_11565 | IT767_11565 | ABC transporter ATP-binding protein | 2.110621 |
| sRNA0361 |  |  | 2.091817 |
| sRNA0270 |  |  | 2.091817 |
| sRNA0285 |  |  | 2.091817 |
| sRNA0008 |  |  | 2.091817 |
| sRNA0309 |  |  | 2.091817 |
| sRNA0295 |  |  | 2.091817 |
| sRNA0241 |  |  | 2.091817 |
| IT767_00745 | eutL | ethanolamine utilization microcompartment protein EutL | 2.071693 |
| sRNA0341 |  |  | 2.064351 |
| sRNA0129 |  |  | 2.059145 |
| sRNA0210 |  |  | 2.032694 |
| IT767_16515 | IT767_16515 | lipocalin family protein | 2.02304 |
| IT767_00740 | eutC | ethanolamine ammonia-lyase subunit EutC | 2.02253 |
| IT767_14300 | IT767_14300 | fimbrial chaperone EcpB | 2.001866 |
| IT767_25165 | IT767_25165 | respiratory chain complex I subunit 1 family protein | -2.01911 |
| IT767_25155 | IT767_25155 | 4Fe-4S dicluster domain-containing protein | -2.04288 |
| IT767_10410 | IT767_10410 | PTS fructose transporter subunit EIIC | -2.16132 |
| IT767_04260 | narJ | nitrate reductase molybdenum cofactor assembly chaperone | -2.18864 |
| IT767_25160 | hycC | formate hydrogenlyase subunit 3 | -2.2043 |
| IT767_24240 | IT767_24240 | sugar ABC transporter permease | -2.55036 |
| IT767_04265 | narI | respiratory nitrate reductase subunit gamma | -2.67501 |
| sRNA0359 |  |  | -4.3765 |
| IT767_23080 | qseB | two-component system response regulator QseB | -8.14729 |
