# Supplementary material for: The Role of the Two-Component QseBC Signaling System in Biofilm Formation and Virulence of Hypervirulent Klebsiella pneumoniae ATCC43816
Source: Front Microbiol. 2022 Apr 6;13:817494. doi: 10.3389/fmicb.2022.817494 (PMC9019566; doi:10.3389/fmicb.2022.817494)
Supplement: Supplementary file 4 [file Table_4.docx]

| **Table S4 Differentially expressed genes in Δ*qseC* relative to the wild-type strain ATCC43816** | | | |
| --- | --- | --- | --- |
| Gene_id | Gene name | Gene description | Log_2_FC |
| sRNA0389 |  |  | 8.699164 |
| IT767_23090 | IT767_23090 | AraC family transcriptional regulator | 8.252966 |
| IT767_23085 | IT767_23085 | YgiW/YdeI family stress tolerance OB fold protein | 7.91659 |
| sRNA0388 |  |  | 6.934268 |
| IT767_23080 | qseB | two-component system response regulator QseB | 5.309403 |
| sRNA0293 |  |  | 5.200914 |
| IT767_02695 | IT767_02695 | DUF2857 domain-containing protein | 5.126913 |
| IT767_21320 | glpD | glycerol-3-phosphate dehydrogenase | 4.675408 |
| sRNA0341 |  |  | 4.545742 |
| IT767_17040 | IT767_17040 | hypothetical protein | 4.463948 |
| IT767_14325 | phnE | phosphonate ABC transporter%2C permease protein PhnE | 4.402547 |
| sRNA0177 |  |  | 4.338417 |
| IT767_16750 | IT767_16750 | hypothetical protein | 3.84477 |
| IT767_11565 | IT767_11565 | ABC transporter ATP-binding protein | 3.511022 |
| IT767_10170 | IT767_10170 | hypothetical protein | 3.44667 |
| sRNA0129 |  |  | 3.406232 |
| IT767_06915 | IT767_06915 | ABC transporter permease | 3.402547 |
| IT767_04050 | IT767_04050 | PAAR domain-containing protein | 3.402547 |
| IT767_06905 | IT767_06905 | hypothetical protein | 3.387999 |
| IT767_03910 | IT767_03910 | branched-chain amino acid ABC transporter permease | 3.360112 |
| IT767_11560 | hlyD | secretion protein HlyD | 3.342964 |
| IT767_14425 | IT767_14425 | fimbria/pilus periplasmic chaperone | 3.338417 |
| IT767_12435 | ahpF | alkyl hydroperoxide reductase subunit F | 3.32254 |
| IT767_14540 | gabP | GABA permease | 3.311945 |
| IT767_11790 | IT767_11790 | two-component-system connector protein YcgZ | 3.311945 |
| sRNA0160 |  |  | 3.308493 |
| sRNA0062 |  |  | 3.229483 |
| sRNA0147 |  |  | 3.126913 |
| IT767_11555 | cecR | transcriptional regulator CecR | 3.055142 |
| IT767_06900 | IT767_06900 | M20 family metallopeptidase | 3.04891 |
| IT767_02015 | dalT | D-arabinitol transporter | 3.000001 |
| IT767_22730 | lsrB | autoinducer 2 ABC transporter substrate-binding protein LsrB | 2.999774 |
| IT767_17790 | phnF | phosphonate metabolism transcriptional regulator PhnF | 2.994463 |
| IT767_23070 | IT767_23070 | NAD(P)H-dependent oxidoreductase | 2.977888 |
| IT767_13260 | IT767_13260 | DUF1471 domain-containing protein | 2.972775 |
| IT767_06280 | IT767_06280 | hypothetical protein | 2.953186 |
| IT767_09575 | IT767_09575 | hypothetical protein | 2.952986 |
| sRNA0194 |  |  | 2.945075 |
| IT767_22735 | lsrF | 3-hydroxy-5-phosphonooxypentane-2%2C4-dione thiolase | 2.930164 |
| IT767_05485 | IT767_05485 | Bcr/CflA family multidrug efflux MFS transporter | 2.913783 |
| IT767_07625 | IT767_07625 | helix-turn-helix transcriptional regulator | 2.892791 |
| IT767_22740 | lsrG | (4S)-4-hydroxy-5-phosphonooxypentane-2%2C3-dione isomerase | 2.878985 |
| IT767_08845 | IT767_08845 | amidohydrolase | 2.878985 |
| IT767_22835 | IT767_22835 | aquaporin | 2.817585 |
| sRNA0113 |  |  | 2.817585 |
| IT767_11570 | IT767_11570 | ABC transporter permease | 2.798587 |
| IT767_22660 | IT767_22660 | YgjV family protein | 2.771588 |
| sRNA0083 |  |  | 2.733491 |
| IT767_23675 | IT767_23675 | hemolysin III family protein | 2.707933 |
| IT767_07330 | IT767_07330 | hypothetical protein | 2.68634 |
| sRNA0119 |  |  | 2.68634 |
| IT767_18320 | aceK | bifunctional isocitrate dehydrogenase kinase/phosphatase | 2.681029 |
| IT767_11740 | IT767_11740 | universal stress protein | 2.654279 |
| IT767_13090 | IT767_13090 | MoaF N-terminal domain-containing protein | 2.64452 |
| IT767_12665 | fepG | iron-enterobactin ABC transporter permease | 2.642285 |
| IT767_10265 | rutB | pyrimidine utilization protein B | 2.633873 |
| IT767_12975 | IT767_12975 | MFS transporter | 2.615951 |
| IT767_17590 | IT767_17590 | fimbrial protein | 2.615951 |
| IT767_07570 | IT767_07570 | aldolase | 2.615951 |
| IT767_18325 | aceA | isocitrate lyase | 2.600041 |
| IT767_16705 | hxsA | His-Xaa-Ser repeat protein HxsA | 2.593828 |
| IT767_09400 | IT767_09400 | glutathione peroxidase | 2.588644 |
| IT767_03890 | tdcD | propionate kinase | 2.579425 |
| IT767_14525 | lhgO | L-2-hydroxyglutarate oxidase | 2.574794 |
| IT767_10260 | rutA | pyrimidine utilization protein A | 2.570863 |
| IT767_00205 | IT767_00205 | DoxX family protein | 2.55813 |
| IT767_04500 | IT767_04500 | gluconate:proton symporter | 2.556479 |
| IT767_11785 | IT767_11785 | diguanylate phosphodiesterase | 2.547392 |
| IT767_20685 | IT767_20685 | HTH-type transcriptional regulator | 2.543947 |
| IT767_18225 | IT767_18225 | reactive intermediate/imine deaminase | 2.532428 |
| IT767_25020 | IT767_25020 | hemin-degrading factor | 2.522842 |
| IT767_21295 | glgC | glucose-1-phosphate adenylyltransferase | 2.510242 |
| IT767_21290 | glgX | glycogen debranching protein GlgX | 2.500934 |
| IT767_22725 | IT767_22725 | autoinducer 2 import system permease LsrD | 2.501181 |
| IT767_03905 | IT767_03905 | branched-chain amino acid ABC transporter substrate-binding protein | 2.497895 |
| IT767_16285 | IT767_16285 | carbon starvation protein A | 2.48738 |
| IT767_06910 | IT767_06910 | phosphotriesterase-related protein | 2.481021 |
| IT767_16710 | hxsC | His-Xaa-Ser system radical SAM maturase HxsC | 2.45521 |
| IT767_23095 | parC | DNA topoisomerase IV subunit A | 2.431007 |
| IT767_25235 | norV | anaerobic nitric oxide reductase flavorubredoxin | 2.424954 |
| IT767_14520 | csiD | carbon starvation induced protein CsiD | 2.420287 |
| IT767_11775 | IT767_11775 | helix-turn-helix transcriptional regulator | 2.406232 |
| IT767_06150 | catA | catechol 1%2C2-dioxygenase | 2.402547 |
| IT767_24725 | ssuD | FMNH2-dependent alkanesulfonate monooxygenase | 2.402547 |
| IT767_06155 | IT767_06155 | Rieske 2Fe-2S domain-containing protein | 2.402547 |
| IT767_12895 | IT767_12895 | glutamate--cysteine ligase | 2.399242 |
| IT767_11760 | IT767_11760 | hypothetical protein | 2.398938 |
| sRNA0193 |  |  | 2.396834 |
| IT767_21300 | glgA | glycogen synthase GlgA | 2.395638 |
| IT767_07945 | abgT | p-aminobenzoyl-glutamate transporter | 2.389947 |
| IT767_02470 | IT767_02470 | sensor domain-containing diguanylate cyclase | 2.382484 |
| IT767_08645 | IT767_08645 | hypothetical protein | 2.381486 |
| IT767_11770 | IT767_11770 | hypothetical protein | 2.381486 |
| IT767_06120 | IT767_06120 | NarK family nitrate/nitrite MFS transporter | 2.368951 |
| IT767_09385 | yeaG | protein kinase YeaG | 2.367422 |
| IT767_18025 | IT767_18025 | LacI family DNA-binding transcriptional regulator | 2.364412 |
| IT767_03955 | IT767_03955 | substrate-binding domain-containing protein | 2.360112 |
| IT767_11735 | IT767_11735 | cation-transporting P-type ATPase | 2.357033 |
| IT767_16755 | bamE | outer membrane protein assembly factor BamE | 2.352917 |
| sRNA0385 |  |  | 2.352055 |
| IT767_17855 | IT767_17855 | ribose ABC transporter permease | 2.338417 |
| IT767_06750 | IT767_06750 | LysR family transcriptional regulator | 2.338417 |
| IT767_10640 | ssuC | aliphatic sulfonate ABC transporter permease SsuC | 2.338417 |
| IT767_05140 | IT767_05140 | 3-hydroxyacyl-CoA dehydrogenase family protein | 2.32377 |
| sRNA0059 |  |  | 2.316391 |
| IT767_00665 | tal | transaldolase | 2.309062 |
| IT767_19990 | IT767_19990 | aromatic acid/H+ symport family MFS transporter | 2.308973 |
| IT767_11795 | IT767_11795 | hypothetical protein | 2.30525 |
| sRNA0407 |  |  | 2.302197 |
| IT767_11460 | dps | DNA starvation/stationary phase protection protein Dps | 2.301966 |
| IT767_16715 | hxsB | His-Xaa-Ser system radical SAM maturase HxsB | 2.292024 |
| IT767_22655 | IT767_22655 | altronate dehydratase | 2.287757 |
| IT767_08665 | IT767_08665 | DUF3131 domain-containing protein | 2.278392 |
| IT767_18140 | IT767_18140 | hypothetical protein | 2.27041 |
| IT767_19610 | pstS | phosphate ABC transporter substrate-binding protein PstS | 2.269769 |
| IT767_23910 | IT767_23910 | DHA2 family efflux MFS transporter permease subunit | 2.261455 |
| IT767_09390 | IT767_09390 | YeaH/YhbH family protein | 2.25629 |
| IT767_03885 | tdcC | threonine/serine transporter TdcC | 2.252444 |
| IT767_12490 | IT767_12490 | sugar ABC transporter ATP-binding protein | 2.248219 |
| IT767_20900 | IT767_20900 | AsmA family protein | 2.247524 |
| IT767_03920 | IT767_03920 | ATP-binding cassette domain-containing protein | 2.244635 |
| IT767_14360 | phoE | phosphoporin PhoE | 2.242108 |
| IT767_09605 | IT767_09605 | general stress protein | 2.242284 |
| IT767_14190 | tauB | taurine ABC transporter ATP-binding subunit | 2.236537 |
| IT767_17955 | IT767_17955 | CidA/LrgA family protein | 2.229483 |
| IT767_08220 | paaY | phenylacetic acid degradation protein PaaY | 2.22213 |
| IT767_10475 | IT767_10475 | glycogen/starch/alpha-glucan phosphorylase | 2.218206 |
| sRNA0213 |  |  | 2.20454 |
| IT767_03365 | IT767_03365 | MFS transporter | 2.200914 |
| IT767_20620 | IT767_20620 | xylose ABC transporter ATP-binding protein | 2.200914 |
| IT767_24220 | IT767_24220 | carbohydrate porin | 2.200914 |
| IT767_13810 | IT767_13810 | shikimate dehydrogenase | 2.200914 |
| IT767_25285 | IT767_25285 | ATP-binding cassette domain-containing protein | 2.200914 |
| IT767_09450 | IT767_09450 | glycosyltransferase family 9 protein | 2.176666 |
| IT767_17860 | IT767_17860 | ABC transporter substrate-binding protein | 2.176666 |
| IT767_22605 | IT767_22605 | DoxX family protein | 2.173169 |
| IT767_03785 | IT767_03785 | SpoVR family protein | 2.167927 |
| sRNA0032 |  |  | 2.159093 |
| IT767_08640 | IT767_08640 | response regulator | 2.153992 |
| IT767_07225 | hpxE | molybdenum cofactor-independent xanthine hydroxylase subunit HpxE | 2.152004 |
| IT767_03895 | pflB | formate C-acetyltransferase | 2.147474 |
| IT767_11800 | IT767_11800 | two-component-system connector protein AriR | 2.14202 |
| IT767_18135 | IT767_18135 | CsbD family protein | 2.141602 |
| IT767_14225 | eutC | ethanolamine ammonia-lyase subunit EutC | 2.13772 |
| IT767_21605 | IT767_21605 | YheV family putative metal-binding protein | 2.137138 |
| IT767_01980 | IT767_01980 | MFS transporter | 2.134242 |
| IT767_22705 | lsrK | autoinducer-2 kinase | 2.128599 |
| IT767_25415 | proW | glycine betaine/L-proline ABC transporter permease ProW | 2.128157 |
| sRNA0396 |  |  | 2.124678 |
| IT767_25295 | IT767_25295 | metal ABC transporter substrate-binding protein | 2.119994 |
| sRNA0157 |  |  | 2.101378 |
| IT767_22115 | IT767_22115 | oxalacetate decarboxylase subunit beta | 2.098256 |
| IT767_06370 | IT767_06370 | ABC transporter substrate-binding protein | 2.094714 |
| IT767_14280 | IT767_14280 | amino acid ABC transporter permease | 2.085436 |
| IT767_14430 | IT767_14430 | type 1 fimbrial protein | 2.071631 |
| IT767_10630 | IT767_10630 | sulfonate ABC transporter substrate-binding protein | 2.071631 |
| IT767_02480 | IT767_02480 | hypothetical protein | 2.069564 |
| IT767_17225 | IT767_17225 | beta-galactosidase | 2.06341 |
| IT767_11875 | IT767_11875 | YbgS-like family protein | 2.057803 |
| IT767_07055 | IT767_07055 | MFS transporter | 2.052317 |
| IT767_00660 | tkt | transketolase | 2.042277 |
| IT767_21305 | glgP | glycogen phosphorylase | 2.041416 |
| IT767_14110 | IT767_14110 | hypothetical protein | 2.04014 |
| IT767_03120 | IT767_03120 | SGNH/GDSL hydrolase family protein | 2.037683 |
| IT767_21285 | glgB | 1%2C4-alpha-glucan branching enzyme | 2.036869 |
| sRNA0093 |  |  | 2.034264 |
| IT767_04585 | IT767_04585 | GlsB/YeaQ/YmgE family stress response membrane protein | 2.032336 |
| IT767_14050 | phoB | phosphate response regulator transcription factor PhoB | 2.032179 |
| IT767_14015 | IT767_14015 | antibiotic biosynthesis monooxygenase | 2.025827 |
| IT767_24235 | IT767_24235 | carbohydrate ABC transporter permease | 2.025827 |
| IT767_23020 | ygiD | 4%2C5-DOPA dioxygenase extradiol | 2.024551 |
| IT767_05560 | sodC | superoxide dismutase [Cu-Zn] SodC2 | 2.023809 |
| IT767_25475 | IT767_25475 | hypothetical protein | 2.02293 |
| IT767_09585 | IT767_09585 | cytochrome ubiquinol oxidase subunit I | 2.019646 |
| IT767_20780 | dppC | dipeptide ABC transporter permease DppC | 2.019307 |
| IT767_19615 | pstC | phosphate ABC transporter permease PstC | 2.018312 |
| IT767_04915 | IT767_04915 | VOC family protein | 2.016489 |
| IT767_12735 | IT767_12735 | transketolase family protein | 2.008268 |
| IT767_20140 | IT767_20140 | NAD(P)-dependent alcohol dehydrogenase | 2.006519 |
| sRNA0285 |  |  | 2.002141 |
| sRNA0295 |  |  | 2.002141 |
| sRNA0309 |  |  | 2.002141 |
| sRNA0241 |  |  | 2.002141 |
| sRNA0008 |  |  | 2.002141 |
| sRNA0270 |  |  | 2.002141 |
| sRNA0361 |  |  | 2.002141 |
| IT767_13245 | cadA | lysine decarboxylase CadA | -2.04285 |
| IT767_09285 | IT767_09285 | sulfurtransferase | -2.05279 |
| IT767_17055 | nrdG | anaerobic ribonucleoside-triphosphate reductase-activating protein | -2.10443 |
| IT767_25140 | hypB | hydrogenase nickel incorporation protein HypB | -2.14043 |
| IT767_10840 | dmsA | dimethylsulfoxide reductase subunit A | -2.22341 |
| IT767_22185 | IT767_22185 | TIGR01212 family radical SAM protein | -2.22809 |
| IT767_10815 | pflB | formate C-acetyltransferase | -2.25505 |
| IT767_04340 | adhE | bifunctional acetaldehyde-CoA/alcohol dehydrogenase | -2.27157 |
| IT767_25155 | IT767_25155 | 4Fe-4S dicluster domain-containing protein | -2.27559 |
| IT767_21510 | cobA | uroporphyrinogen-III C-methyltransferase | -2.2883 |
| IT767_25150 | hycA | formate hydrogenlyase regulator HycA | -2.30766 |
| sRNA0156 |  |  | -2.39403 |
| IT767_21015 | nikC | nickel ABC transporter permease subunit NikC | -2.45094 |
| IT767_21520 | IT767_21520 | nitrite reductase large subunit | -2.48583 |
| IT767_20490 | gpmM | 2%2C3-bisphosphoglycerate-independent phosphoglycerate mutase | -2.6014 |
| IT767_16465 | IT767_16465 | cupin domain-containing protein | -2.66552 |
| IT767_22470 | IT767_22470 | U32 family peptidase | -2.67121 |
| IT767_01435 | glpC | anaerobic glycerol-3-phosphate dehydrogenase subunit C | -2.80264 |
| IT767_07860 | IT767_07860 | FAD:protein FMN transferase | -2.84282 |
| IT767_22475 | IT767_22475 | U32 family peptidase | -2.8936 |
| IT767_17050 | nrdD | anaerobic ribonucleoside-triphosphate reductase | -2.90009 |
| IT767_21515 | nirD | nitrite reductase small subunit NirD | -3.18527 |
| IT767_04265 | narI | respiratory nitrate reductase subunit gamma | -3.44402 |
| IT767_04255 | narH | nitrate reductase subunit beta | -3.63155 |
| IT767_04260 | narJ | nitrate reductase molybdenum cofactor assembly chaperone | -3.62929 |
| IT767_07870 | IT767_07870 | anion permease | -3.8379 |
| IT767_07865 | IT767_07865 | flavocytochrome c | -4.18899 |
| IT767_07875 | IT767_07875 | class I fumarate hydratase | -4.36887 |
| IT767_23075 | qseC | two-component system sensor histidine kinase QseC | -7.77923 |
| sRNA0359 |  |  | -7.97804 |
