# Supplementary material for: The Role of the Two-Component QseBC Signaling System in Biofilm Formation and Virulence of Hypervirulent Klebsiella pneumoniae ATCC43816
Source: Front Microbiol. 2022 Apr 6;13:817494. doi: 10.3389/fmicb.2022.817494 (PMC9019566; doi:10.3389/fmicb.2022.817494)
Supplement: Supplementary file 5 [file Table_5.docx]

| **Table S5 Differentially expressed genes in Δ*qseBC* relative to the wild-type strain ATCC43816** | | | |
| --- | --- | --- | --- |
| Gene_id | Gene name | Gene description | Log_2_FC |
| sRNA0293 |  |  | 4.650717307 |
| IT767_00735 | eutB | ethanolamine ammonia-lyase subunit alpha | 4.622702931 |
| sRNA0177 |  |  | 4.53524009 |
| IT767_00730 | eutA | ethanolamine ammonia-lyase reactivating factor EutA | 4.489091629 |
| IT767_00740 | eutC | ethanolamine ammonia-lyase subunit EutC | 4.041131019 |
| IT767_25265 | srlB | PTS glucitol/sorbitol transporter subunit IIA | 3.950277589 |
| IT767_14425 | IT767_14425 | fimbria/pilus periplasmic chaperone | 3.904473899 |
| IT767_18225 | IT767_18225 | reactive intermediate/imine deaminase | 3.892792094 |
| IT767_14540 | gabP | GABA permease | 3.435704416 |
| IT767_00725 | eutH | ethanolamine utilization protein EutH | 3.429318957 |
| IT767_00750 | eutK | ethanolamine utilization microcompartment protein EutK | 3.350208196 |
| IT767_00720 | eutG | ethanolamine utilization ethanol dehydrogenase EutG | 3.323649302 |
| IT767_00745 | eutL | ethanolamine utilization microcompartment protein EutL | 3.279401185 |
| IT767_18055 | IT767_18055 | helix-turn-helix transcriptional regulator | 3.272205684 |
| IT767_00755 | eutR | HTH-type transcriptional regulator EutR | 3.131408045 |
| IT767_11570 | IT767_11570 | ABC transporter permease | 3.092994632 |
| IT767_00715 | eutJ | ethanolamine utilization protein EutJ | 3.072091935 |
| IT767_18220 | IT767_18220 | AzlD domain-containing protein | 3.065754806 |
| IT767_11565 | IT767_11565 | ABC transporter ATP-binding protein | 3.042377292 |
| IT767_06320 | IT767_06320 | ferritin-like domain-containing protein | 3.04030979 |
| IT767_20840 | bcsF | cellulose biosynthesis protein BcsF | 3.03774043 |
| IT767_05350 | IT767_05350 | ABC transporter permease | 3.03774043 |
| IT767_16170 | IT767_16170 | 2-dehydro-3-deoxygalactonokinase | 3.03774043 |
| IT767_16515 | IT767_16515 | lipocalin family protein | 2.96953055 |
| sRNA0129 |  |  | 2.907208867 |
| IT767_18135 | IT767_18135 | CsbD family protein | 2.874760221 |
| IT767_15960 | IT767_15960 | DUF1328 domain-containing protein | 2.868056501 |
| IT767_14525 | lhgO | L-2-hydroxyglutarate oxidase | 2.859170538 |
| sRNA0119 |  |  | 2.808258584 |
| IT767_18140 | IT767_18140 | hypothetical protein | 2.792015505 |
| IT767_11560 | hlyD | secretion protein HlyD | 2.785977137 |
| IT767_16570 | IT767_16570 | carbohydrate porin | 2.774647294 |
| sRNA0407 |  |  | 2.757632511 |
| IT767_08665 | IT767_08665 | DUF3131 domain-containing protein | 2.754074692 |
| IT767_13700 | IT767_13700 | PLP-dependent cysteine synthase family protein | 2.742202471 |
| IT767_15965 | osmY | molecular chaperone OsmY | 2.684514582 |
| IT767_13665 | IT767_13665 | YbaY family lipoprotein | 2.676083475 |
| IT767_14400 | IT767_14400 | hypothetical protein | 2.675351287 |
| IT767_06315 | IT767_06315 | hypothetical protein | 2.675063059 |
| IT767_02015 | dalT | D-arabinitol transporter | 2.672743613 |
| IT767_00205 | IT767_00205 | DoxX family protein | 2.655992249 |
| IT767_09600 | IT767_09600 | ferritin-like domain-containing protein | 2.650717307 |
| IT767_14395 | IT767_14395 | winged helix-turn-helix domain-containing protein | 2.64480219 |
| IT767_20835 | bcsG | cellulose biosynthesis protein BcsG | 2.643676322 |
| IT767_18910 | IT767_18910 | amidohydrolase family protein | 2.628349494 |
| IT767_16705 | hxsA | His-Xaa-Ser repeat protein HxsA | 2.626387978 |
| IT767_10475 | IT767_10475 | glycogen/starch/alpha-glucan phosphorylase | 2.625816557 |
| IT767_02470 | IT767_02470 | sensor domain-containing diguanylate cyclase | 2.61770225 |
| sRNA0059 |  |  | 2.606901075 |
| IT767_16305 | IT767_16305 | NAD-dependent succinate-semialdehyde dehydrogenase | 2.606342836 |
| IT767_18215 | IT767_18215 | AzlC family ABC transporter permease | 2.600828431 |
| IT767_16860 | IT767_16860 | sugar phosphate isomerase/epimerase | 2.594133779 |
| IT767_21300 | glgA | glycogen synthase GlgA | 2.586503818 |
| IT767_11980 | cydX | cytochrome bd-I oxidase subunit CydX | 2.582545805 |
| IT767_12895 | IT767_12895 | glutamate--cysteine ligase | 2.572714795 |
| IT767_16490 | IT767_16490 | efflux transporter outer membrane subunit | 2.569187422 |
| IT767_06280 | IT767_06280 | hypothetical protein | 2.556456576 |
| IT767_08660 | IT767_08660 | DUF3131 domain-containing protein | 2.542975739 |
| IT767_12490 | IT767_12490 | sugar ABC transporter ATP-binding protein | 2.53524009 |
| IT767_06915 | IT767_06915 | ABC transporter permease | 2.53524009 |
| IT767_20900 | IT767_20900 | AsmA family protein | 2.533444576 |
| IT767_09225 | astE | succinylglutamate desuccinylase | 2.528545437 |
| IT767_08645 | IT767_08645 | hypothetical protein | 2.509973331 |
| IT767_05485 | IT767_05485 | Bcr/CflA family multidrug efflux MFS transporter | 2.493880246 |
| sRNA0319 |  |  | 2.49084597 |
| IT767_08640 | IT767_08640 | response regulator | 2.486330489 |
| IT767_14520 | csiD | carbon starvation induced protein CsiD | 2.481468833 |
| IT767_11555 | cecR | transcriptional regulator CecR | 2.477906915 |
| IT767_11585 | IT767_11585 | endonuclease/exonuclease/phosphatase family protein | 2.477302644 |
| IT767_24725 | ssuD | FMNH2-dependent alkanesulfonate monooxygenase | 2.473839545 |
| IT767_21305 | glgP | glycogen phosphorylase | 2.473514504 |
| IT767_10170 | IT767_10170 | hypothetical protein | 2.464850762 |
| IT767_14430 | IT767_14430 | type 1 fimbrial protein | 2.464850762 |
| IT767_14295 | IT767_14295 | CS1-pili formation C-terminal domain-containing protein | 2.44729239 |
| IT767_06905 | IT767_06905 | hypothetical protein | 2.43966243 |
| IT767_12460 | IT767_12460 | ParB-like nuclease domain-containing protein | 2.435704416 |
| IT767_25455 | IT767_25455 | DUF883 domain-containing protein | 2.435274979 |
| IT767_22765 | dhaM | dihydroxyacetone kinase subunit DhaM | 2.432670356 |
| IT767_19990 | IT767_19990 | aromatic acid/H+ symport family MFS transporter | 2.431404279 |
| IT767_19835 | dsdX | D-serine transporter DsdX | 2.431404279 |
| IT767_06335 | IT767_06335 | SDR family oxidoreductase | 2.421583308 |
| IT767_04490 | IT767_04490 | D-amino acid dehydrogenase | 2.417467106 |
| IT767_18125 | IT767_18125 | cupin domain-containing protein | 2.414103652 |
| IT767_13260 | IT767_13260 | DUF1471 domain-containing protein | 2.411592376 |
| IT767_22770 | dhaL | dihydroxyacetone kinase ADP-binding subunit DhaL | 2.401973559 |
| sRNA0325 |  |  | 2.395062432 |
| IT767_10630 | IT767_10630 | sulfonate ABC transporter substrate-binding protein | 2.39085018 |
| IT767_22500 | IT767_22500 | type 1 glutamine amidotransferase | 2.389019349 |
| IT767_20685 | IT767_20685 | HTH-type transcriptional regulator | 2.385344659 |
| IT767_04585 | IT767_04585 | GlsB/YeaQ/YmgE family stress response membrane protein | 2.371201931 |
| IT767_11505 | IT767_11505 | DUF1471 domain-containing protein | 2.366726221 |
| IT767_12755 | IT767_12755 | LysR family transcriptional regulator | 2.365315088 |
| sRNA0083 |  |  | 2.356902848 |
| IT767_06420 | IT767_06420 | alpha/beta hydrolase | 2.356269949 |
| IT767_21075 | IT767_21075 | AI-2E family transporter | 2.348519846 |
| IT767_16700 | IT767_16700 | KAP family P-loop domain protein | 2.344868047 |
| IT767_15775 | msyB | acidic protein MsyB | 2.342595012 |
| IT767_00710 | IT767_00710 | aldehyde dehydrogenase EutE | 2.342088536 |
| IT767_20860 | bcsA | UDP-forming cellulose synthase catalytic subunit | 2.339968136 |
| IT767_21295 | glgC | glucose-1-phosphate adenylyltransferase | 2.333606229 |
| IT767_08845 | IT767_08845 | amidohydrolase | 2.328789212 |
| IT767_17860 | IT767_17860 | ABC transporter substrate-binding protein | 2.319511399 |
| IT767_20320 | IT767_20320 | lysophospholipid acyltransferase family protein | 2.312847668 |
| IT767_24055 | IT767_24055 | type 1 fimbrial protein | 2.307829594 |
| IT767_25260 | srlD | sorbitol-6-phosphate dehydrogenase | 2.306421399 |
| IT767_06900 | IT767_06900 | M20 family metallopeptidase | 2.300774836 |
| IT767_22605 | IT767_22605 | DoxX family protein | 2.299427153 |
| IT767_08145 | astD | succinylglutamate-semialdehyde dehydrogenase | 2.298437225 |
| IT767_09235 | astD | succinylglutamate-semialdehyde dehydrogenase | 2.288147228 |
| IT767_09585 | IT767_09585 | cytochrome ubiquinol oxidase subunit I | 2.279141731 |
| IT767_06370 | IT767_06370 | ABC transporter substrate-binding protein | 2.272205684 |
| IT767_23485 | IT767_23485 | MFS transporter | 2.272205684 |
| IT767_24655 | IT767_24655 | YgdI/YgdR family lipoprotein | 2.270653564 |
| IT767_06350 | IT767_06350 | YdeI family stress tolerance OB fold protein | 2.266480696 |
| IT767_09390 | IT767_09390 | YeaH/YhbH family protein | 2.26018077 |
| IT767_16505 | IT767_16505 | CusA/CzcA family heavy metal efflux RND transporter | 2.259030295 |
| IT767_21320 | glpD | glycerol-3-phosphate dehydrogenase | 2.257666621 |
| IT767_05480 | cfa | cyclopropane fatty acyl phospholipid synthase | 2.256349738 |
| IT767_01445 | glpA | anaerobic glycerol-3-phosphate dehydrogenase subunit A | 2.254127018 |
| IT767_10470 | IT767_10470 | STAS domain-containing protein | 2.253726263 |
| IT767_23860 | IT767_23860 | urease accessory protein UreD | 2.251447124 |
| IT767_04975 | casA | type I-E CRISPR-associated protein Cse1/CasA | 2.247958138 |
| sRNA0210 |  |  | 2.246793265 |
| IT767_11590 | clsB | cardiolipin synthase ClsB | 2.238726124 |
| IT767_06115 | IT767_06115 | nitrate reductase subunit alpha | 2.23267732 |
| IT767_08260 | paaK | phenylacetate-CoA oxygenase/reductase subunit PaaK | 2.222082204 |
| IT767_12830 | IT767_12830 | amino acid ABC transporter ATP-binding protein | 2.213311995 |
| IT767_04400 | IT767_04400 | polyisoprenoid-binding protein | 2.209011857 |
| sRNA0396 |  |  | 2.207110656 |
| IT767_01975 | fbaB | class I fructose-bisphosphate aldolase | 2.200556684 |
| IT767_11875 | IT767_11875 | YbgS-like family protein | 2.196876598 |
| IT767_14110 | IT767_14110 | hypothetical protein | 2.191285689 |
| IT767_12435 | ahpF | alkyl hydroperoxide reductase subunit F | 2.178042754 |
| IT767_04985 | cas6e | type I-E CRISPR-associated protein Cas6/Cse3/CasE | 2.164402394 |
| IT767_08135 | astE | succinylglutamate desuccinylase | 2.162328066 |
| IT767_16750 | IT767_16750 | hypothetical protein | 2.159730955 |
| IT767_25470 | stpA | DNA-binding protein StpA | 2.153741701 |
| IT767_06330 | IT767_06330 | alpha-amylase family protein | 2.153341749 |
| IT767_18035 | iraM | anti-adapter protein IraM | 2.149949934 |
| IT767_23920 | evgA | acid-sensing system DNA-binding response regulator EvgA | 2.135833242 |
| IT767_06130 | kmrA | efflux MFS transporter KmrA | 2.125783623 |
| IT767_09385 | yeaG | protein kinase YeaG | 2.12226345 |
| IT767_08140 | astB | N-succinylarginine dihydrolase | 2.122282901 |
| IT767_03910 | IT767_03910 | branched-chain amino acid ABC transporter permease | 2.12020259 |
| sRNA0093 |  |  | 2.105555814 |
| IT767_21290 | glgX | glycogen debranching protein GlgX | 2.104220681 |
| IT767_23910 | IT767_23910 | DHA2 family efflux MFS transporter permease subunit | 2.097118977 |
| IT767_03785 | IT767_03785 | SpoVR family protein | 2.092296594 |
| IT767_06345 | IT767_06345 | hypothetical protein | 2.087781113 |
| IT767_11320 | bssR | biofilm formation regulator BssR | 2.073825849 |
| IT767_06120 | IT767_06120 | NarK family nitrate/nitrite MFS transporter | 2.072268113 |
| IT767_01875 | IT767_01875 | ABC transporter permease | 2.06885571 |
| IT767_03890 | tdcD | propionate kinase | 2.065754806 |
| sRNA0118 |  |  | 2.065754806 |
| IT767_21235 | ggt | gamma-glutamyltransferase | 2.059506659 |
| IT767_02005 | IT767_02005 | mannitol dehydrogenase family protein | 2.057192793 |
| IT767_13090 | IT767_13090 | MoaF N-terminal domain-containing protein | 2.054614249 |
| IT767_17545 | ecnB | lipoprotein toxin entericidin B | 2.049813263 |
| IT767_02465 | IT767_02465 | alpha/beta hydrolase | 2.045558092 |
| IT767_20845 | bcsE | cellulose biosynthesis protein BcsE | 2.039361133 |
| IT767_18210 | IT767_18210 | serine dehydratase subunit alpha family protein | 2.039086856 |
| IT767_25425 | nrdF | class 1b ribonucleoside-diphosphate reductase subunit beta | 2.03774043 |
| IT767_06195 | IT767_06195 | CcdB family protein | 2.03774043 |
| sRNA0198 |  |  | 2.031610068 |
| IT767_08220 | paaY | phenylacetic acid degradation protein PaaY | 2.028280101 |
| IT767_23730 | IT767_23730 | DUF554 domain-containing protein | 2.014407926 |
| IT767_15810 | IT767_15810 | hypothetical protein | 2.013287387 |
| IT767_06355 | IT767_06355 | hypothetical protein | 2.005690709 |
| IT767_20930 | IT767_20930 | alpha%2Calpha-trehalase | 2.002567501 |
| IT767_06305 | IT767_06305 | SDR family oxidoreductase | 2.001671176 |
| IT767_09605 | IT767_09605 | general stress protein | 2.00180789 |
| IT767_25175 | IT767_25175 | formate hydrogenlyase complex iron-sulfur subunit | -2.10181092 |
| IT767_25150 | hycA | formate hydrogenlyase regulator HycA | -2.27211483 |
| IT767_04260 | narJ | nitrate reductase molybdenum cofactor assembly chaperone | -2.27147463 |
| IT767_25170 | hycE | formate hydrogenlyase subunit HycE | -2.39930685 |
| IT767_02990 | IT767_02990 | DUF2560 family protein | -2.41229249 |
| IT767_25155 | IT767_25155 | 4Fe-4S dicluster domain-containing protein | -2.43338652 |
| IT767_25160 | hycC | formate hydrogenlyase subunit 3 | -2.46086033 |
| IT767_25165 | IT767_25165 | respiratory chain complex I subunit 1 family protein | -2.6251306 |
| IT767_03070 | IT767_03070 | phage tail assembly chaperone family protein%2C TAC | -2.67421328 |
| IT767_05075 | IT767_05075 | cytochrome c-type biogenesis protein CcmH | -2.76592944 |
| IT767_04265 | narI | respiratory nitrate reductase subunit gamma | -2.85385343 |
| IT767_03065 | IT767_03065 | phage tail protein | -2.89771932 |
| IT767_02970 | IT767_02970 | phage holin family protein | -3.1086161 |
| IT767_03100 | IT767_03100 | nitrite transporter | -3.27211483 |
| IT767_03025 | IT767_03025 | HK97 family phage prohead protease | -3.38957241 |
| IT767_05465 | asr | acid resistance repetitive basic protein Asr | -3.61188647 |
| IT767_03075 | IT767_03075 | phage tail protein | -3.6935786 |
| IT767_07760 | asr | acid resistance repetitive basic protein Asr | -3.90871399 |
| sRNA0108 |  |  | -4.12011174 |
| sRNA0359 |  |  | -4.37836742 |
| IT767_23075 | qseC | two-component system sensor histidine kinase QseC | -7.70793389 |
| IT767_23080 | qseB | two-component system response regulator QseB | -7.96858565 |
